# Supplementary material for: Outpatient care changes and associated mortality among Veterans with heart failure during the COVID-19 pandemic
Source: PLoS One. 2025 May 16;20(5):e0323308. doi: 10.1371/journal.pone.0323308 (PMC12083836; doi:10.1371/journal.pone.0323308)
Supplement: S1 Data — (DOCX) [file pone.0323308.s001.docx]

**Supplemental Material:**

**Supplement 1:** Types of Clinic Visits Included in the Analysis Identified via Primary Clinic Stop Codes

**Supplement 2:** Primary Care/Cardiology Clinic Visits

**Supplement 3:** Virtual Video Visits and Telephone Visits

**Supplement 4:** Annual Cardiology and Primary Care Specific Outpatient Visit Days (either in-person or video-only) per 100 Veterans with Heart Failure Broken Down by Month from 2018-2020.

**Supplement 5:** Annual Cardiology and Primary Care Specific Video-Only Outpatient Visit Days per 100 Veterans with Heart Failure Broken Down by Month from 2018-2020.

**Supplement 6:** Number and characteristics of Veterans per year for Analysis of Patterns of Annual Outpatient Visits per Veteran and Deaths per 1000 Veterans per year for Veterans with Heart Failure between 2018 and 2020

**Supplement 7:** Complete List of Baseline Characteristics (with list of comorbidities by Elixhauser comorbidity definitions) for the Association of Type of Outpatient Visits with Subsequent Mortality in Patients with Heart Failure

**Supplement 8:** Association of Type of Outpatient Visits with Subsequent Mortality in Patients with Heart Failure Using Historical Cohorts (2018 and 2019)

**Supplement 9:** Sensitivity Analyses for the Association of Type of Outpatient Visits with Subsequent Mortality in Patients with Heart Failure Using a Blanking Period

**Supplement 10:** Sensitivity Analyses for the Association of Type of Outpatient Visits with Subsequent Mortality in Patients with Heart Failure Including Days with Only Primary Care or Cardiology Outpatient Clinical Encounters

**1: Types of Clinic Visits Included in the Analysis Identified via Primary Clinic Stop Codes**

| **Description** |
| --- |
| ADULT DAY HEALTH CARE |
| ALLERGY IMMUNOLOGY |
| AMPUTATION CLINIC |
| AMYOTROPHIC LATERAL SCLEROSIS (ALS) CENTER |
| ANESTHESIA CONSULT, INCLUDING PRE-PROCEDURE AND EXPANDED POST-PROCEDURE ASSESSMENT |
| ANESTHESIA SPECIAL PROCEDURES IN OPERATING ROOM SUITE OR A NON-OPERATING ROOM PROCEDURE ROOM |
| ANTI-COAGULATION CLINIC |
| ASSISTED HEMODIALYSIS |
| AUDIOLOGY |
| BARIATRIC SURGERY |
| BLIND REHABILITATION CENTER |
| BLIND REHABILITATION OUTPATIENT SPECIALIST |
| CARDIAC CATHETERIZATION |
| CARDIAC IMPLANTABLE ELECTRONIC DEVICES (CIED) |
| CARDIAC STRESS TEST |
| CARDIAC SURGERY |
| CARDIO-PULMONARY REHABILITATION |
| CARDIOLOGY |
| CARDIOTHORACIC SURGERY |
| CARE OF HOME TELEHEALTH PROGRAM PATIENTS |
| CHAPLAIN SERVICE - GROUP |
| CHAPLAIN SERVICE - INDIVIDUAL |
| CHEMOTHERAPY PROCEDURES UNIT – MEDICINE |
| CHIROPRACTIC CARE |
| COMMUNITY ADULT DAY HEALTH CARE FOLLOW-UP |
| COMMUNITY CARE CONSULT |
| COMMUNITY RESIDENTIAL CARE (CRC) |
| COMPLEMENTARY AND INTEGRATIVE HEALTH TREATMENT |
| COMPREHENSIVE WOMEN’S PRIMARY CARE CLINIC |
| CONTINUOUS AMBULATORY PERITONEAL DIALYSIS |
| CYSTOSCOPY ROOM UNIT FOR OUTPATIENT |
| DENTAL |
| DEPARTMENT OF DEFENSE NON-VA CARE |
| DEPARTMENT OF HOUSING AND URBAN DEVELOPMENT AND VETERANS’ AFFAIRS SUPPORTIVE HOUSING PROGRAM- INDIVIDUAL |
| DEPARTMENT OF HOUSING AND URBAN DEVELOPMENT AND VETERANS’ AFFAIRS SUPPORTIVE HOUSING PROGRAM GROUP |
| DERMATOLOGY |
| DIABETES |
| ELECTROPHYSIOLOGY LABORATORY |
| ENDOCRINOLOGY |
| EPILEPSY CENTER OF EXCELLENCE |
| EYE TELEHEALTH SCREENING |
| GASTROENTEROLOGY |
| GASTROENTEROLOGY ENDOSCOPY |
| GENERAL INTERNAL MEDICINE |
| GENERAL SURGERY |
| GENOMIC CARE |
| GERIATRIC PATIENT-ALIGNED CARE TEAM |
| GERIATRIC PROBLEM-FOCUSED CONSULTATION CLINIC |
| GEROPSYCHIATRICS - INDIVIDUAL |
| GEROPSYCHIATRICS GROUP |
| GYNECOLOGY |
| HAND SURGERY |
| HEALTH AND WELL-BEING SERVICES |
| HEALTH CARE FOR HOMELESS VETERANS/HOMELESS CHRONICALLY MENTALLY ILL |
| HEALTH SCREENING |
| HEMATOLOGY |
| HEPATOLOGY CLINIC |
| HOME AND COMMUNITY-BASED CARE (HCBC) ASSESSMENT |
| HOME TREATMENT SERVICES- (NON-HOME-BASED PRIMARY CARE, NON-HOSPITAL-IN-HOME, NON-HOME-AND-COMMUNITY-BASED SERVICES) |
| HOME-BASED PRIMARY CARE - CLINICAL PHARMACIST |
| HOME-BASED PRIMARY CARE - DIETITIAN |
| HOME-BASED PRIMARY CARE - NURSE EXTENDER |
| HOME-BASED PRIMARY CARE - OTHER |
| HOME-BASED PRIMARY CARE - PHYSICIAN |
| HOME-BASED PRIMARY CARE - PSYCHIATRIST |
| HOME-BASED PRIMARY CARE - PSYCHOLOGIST |
| HOME-BASED PRIMARY CARE - SOCIAL WORKER |
| HOME-BASED PRIMARY CARE - THERAPIST |
| HOME-BASED PRIMARY CARE – REGISTERED NURSE OR LICENSED PRACTICAL NURSE |
| HOME/SELF CONTINUOUS PERITONEAL DIALYSIS TRAINING |
| HOME/SELF HEMODIALYSIS TRAINING |
| HOSPITAL AT HOME |
| HYPERTENSION |
| INFECTIOUS DISEASE |
| INTENSIVE COMMUNITY MENTAL HEALTH RECOVERY SERVICES (ICMHR)-INDIVIDUAL |
| INTERVENTIONAL RADIOLOGY (IR) PROCEDURE |
| INTERVENTIONAL RADIOLOGY CLINIC |
| KINESIOTHERAPY |
| LIMITED SELF CARE CONTINUOUS PERITONEAL DIALYSIS |
| LIMITED SELF CARE HEMODIALYSIS |
| LOW VISION CARE |
| MEDICAL PHYSICIAN (MD) PERFORM INVASIVE OPERATING ROOM PROCEDURE |
| MEDICAL PRE-PROCEDURE EVALUATION |
| MEDICAL PROCEDURE UNIT |
| MEDICAL SPECIALTY SHARED APPOINTMENT |
| MEDICAL/SURGICAL DAY UNIT |
| MENTAL HEALTH CLINIC - GROUP |
| MENTAL HEALTH CLINIC INDIVIDUAL |
| MENTAL HEALTH COMPENSATED WORK THERAPY/SUPPORTED EMPLOYMENT |
| MENTAL HEALTH COMPENSATED WORK THERAPY/TRANSITIONAL WORK EXPERIENCE |
| MENTAL HEALTH INTEGRATED CARE GROUP |
| MENTAL HEALTH INTEGRATED CARE INDIVIDUAL |
| MENTAL HEALTH INTERVENTION BIOMEDICAL – INDIVIDUAL |
| MENTAL HEALTH INTERVENTION BIOMEDICAL CARE GROUP |
| MENTAL HEALTH RISK FACTOR REDUCTION GROUP |
| MENTAL HEALTH TEAM CASE MANAGEMENT |
| MENTAL HEALTH VOCATIONAL ASSISTANCE INDIVIDUAL |
| MULTIPLE SCLEROSIS (MS) |
| MYOCARDIAL PERFUSION |
| NEUROLOGY |
| NEUROSURGERY |
| NON-OPERATING ROOM ANESTHESIA PROCEDURE |
| NUTRITION/DIETETICS -GROUP |
| NUTRITION/DIETETICS-INDIVIDUAL |
| OBSTETRICS |
| OCCUPATIONAL HEALTH |
| OCCUPATIONAL THERAPY |
| ONCOLOGY/TUMOR |
| OPHTHALMOLOGY |
| OPIOID TREATMENT PROGRAM |
| OPTOMETRY |
| ORTHOPEDICS/JOINT SURGERY |
| OTOLARYNGOLOGY (ENT) |
| PAIN CLINIC |
| PALLIATIVE CARE |
| PARKINSON’S DISEASE RESEARCH, EDUCATION, AND CLINICAL CENTERS |
| PATIENT CARE IN OPERATING ROOM |
| PHYSICAL MEDICINE AND REHABILITATION AMPUTATION CLINIC |
| PHYSICAL MEDICINE AND REHABILITATION PHYSICIAN |
| PHYSICAL THERAPY |
| PLASTIC SURGERY |
| POLYTRAUMA/TRAUMATIC BRAIN INJURY -INDIVIDUAL |
| POST-TRAUMATIC STRESS DISORDER OUTPATIENT SPECIALTY & RESIDENTIAL PROGRAMS: GROUP |
| PRE-BED CARE (MEDICAL SERVICE) |
| PRE-SURGERY EVALUATION |
| PRIMARY CARE SHARED APPOINTMENT |
| PRIMARY CARE/MEDICINE |
| PROSTHETIC & SENSORY AIDS SERVICE |
| PSYCHIATRY |
| PSYCHOLOGICAL TESTING |
| PSYCHOSOCIAL REHABILITATION AND RECOVERY CENTER GROUP |
| PSYCHOSOCIAL REHABILITATION AND RECOVERY INDIVIDUAL |
| PTSD OUTPATIENT SPECIALTY & RESIDENTIAL PROGRAMS: INDIVIDUAL |
| PULMONARY/CHEST |
| RADIATION ONCOLOGY (RADIATION THERAPY) |
| RECREATION THERAPY SERVICE |
| REHABILITATION SERVICES GROUP |
| RENAL/NEPHROLOGY (EXCEPT DIALYSIS) |
| RESIDENTIAL REHABILITATION ADMISSION SCREENING SERVICES |
| RESIDENTIAL REHABILITATION TREATMENT PROGRAM (RRTP) OUTPATIENT – INDIVIDUAL |
| RESIDENTIAL REHABILITATION TREATMENT PROGRAM (RRTP)- GROUP |
| RESIDENTIAL REHABILITATION TREATMENT PROGRAM (RRTP)- INDIVIDUAL |
| RESPIRATORY THERAPY |
| RHEUMATOLOGY/ARTHRITIS |
| SLEEP MEDICINE |
| SOCIAL WORK SERVICE |
| SPEECH LANGUAGE PATHOLOGY |
| SPINAL CORD INJURY |
| SPINAL CORD INJURY HOME CARE PROGRAM |
| SPINAL CORD INJURY TELEHEALTH AND VIRTUAL CARE |
| SPINAL SURGERY |
| SUBSTANCE USE DISORDER GROUP |
| SUBSTANCE USE DISORDER INDIVIDUAL |
| SURGICAL ONCOLOGY |
| SURGICAL PROCEDURE UNIT |
| TELEPHONE/CHAPLAIN |
| THORACIC SURGERY |
| UROLOGY |
| VETERANS AFFAIRS-REFER TO HOME AND COMMUNITY-BASED SERVICES PROVIDERS |
| VASCULAR SURGERY |
| VETERANS JUSTICE OUTREACH |
| VISION IMPAIRMENT SERVICES IN OUTPATIENT REHABILITATION & ADVANCED BLIND REHABILTATION |
| VIST COORDINATOR |
| VISUAL IMPAIRMENT CENTER TO OPTIMIZE REMAINING SIGHT (VICTORS) & ADVANCED LOW VISION |
| WEIGHT MANAGEMENT AND MOVE! PROGRAM- GROUP |
| WEIGHT MANAGEMENT AND MOVE! PROGRAM- INDIVIDUAL |
| WHEELCHAIR AND ADVANCED MOBILITY CLINIC |
| WOMEN GENDER SPECIFIC PREVENTIVE CARE |
| WOUND TREATMENT & OSTOMY CARE |

**2: Primary Care/Cardiology Clinic Visits**

| **Description** |
| --- |
| CARDIAC CATHETERIZATION |
| CARDIAC IMPLANTABLE ELECTRONIC DEVICES (CIED) |
| CARDIAC STRESS TEST |
| CARDIAC SURGERY |
| CARDIO-PULMONARY REHABILITATION |
| CARDIOLOGY |
| CARDIOTHORACIC SURGERY |
| COMPREHENSIVE WOMEN’S PRIMARY CARE CLINIC |
| ELECTROPHYSIOLOGY LABORATORY |
| GENERAL INTERNAL MEDICINE |
| GERIATRIC PATIENT-ALIGNED CARE TEAM |
| GERIATRIC PROBLEM-FOCUSED CONSULTATION CLINIC |
| GEROPSYCHIATRICS GROUP |
| HOME-BASED PRIMARY CARE - CLINICAL PHARMACIST |
| HOME-BASED PRIMARY CARE - PHYSICIAN |
| HOME-BASED PRIMARY CARE – REGISTERED NURSE OR LICENSED PRACTICAL NURSE |
| HOSPITAL AT HOME |
| HYPERTENSION |
| MYOCARDIAL PERFUSION |
| OBSTETRICS |
| PRIMARY CARE SHARED APPOINTMENT |
| PRIMARY CARE/MEDICINE |
| WOMEN GENDER SPECIFIC PREVENTIVE CARE |

**3: Virtual Video Visits and Telephone Visits**

**Secondary Clinic stop codes are used to distinguish in-person versus remote Video-telehealth visits within the same primary stop code:**

| Telehealth Type | Secondary Stop Codes |
| --- | --- |
| Clinical Video Telehealth (Patient Site) | 136, 444, 446, 490, 644, 690, 723 |
| Clinical Video Telehealth (Provider Site) | 137, 440, 445, 447, 491, 645, 692, 693, 708, 724 |
| Clinical Video Telehealth (Provider to Off-Site Patient) | 179, 648, 679 |
| Clinical Video Telehealth (Provider to ER/Emergent) | 699 |
| Store and Forward Telehealth (Patient Site) | 646, 694 |
| Store and Forward Telehealth (Provider Site) | 647, 695, 696 |
| Store and Forward Telehealth (Provider to Off-Site Patient) | 189,698 |

**Telephone Only Visits Identified by Primary Clinic Stop Codes:**

| Description |
| --- |
| TELEPHONE PRIMARY CARE |
| TELEPHONE/MEDICINE |
| TELEPHONE/ANCILLARY |
| CLINICAL PHARMACY |
| TELEPHONE TRIAGE |
| TELEPHONE CONTACT BY HT STAFF |
| TELEPHONE/PSYCH |
| HBPC/TELEPHONE |
| HT NON-VIDEO MONITORING |
| TELEPHONE SURGERY |
| TELEPHONE/HUD VASH |
| TELEPHONE/REHAB AND SUPPORT |
| TELEPHONE/GERIATRICS |
| TELEPHONE/SUB USE DISORDER |
| TELE CASE MGMT |
| TELEPHONE/OPTOMETRY |
| TELEPHONE/NEUROLOGY |
| TELEPHONE/DIAGNOSTIC |
| TELEPHONE INTENSIVE COMMUNITY MENTAL HEALTH RECOVERY SERVICES (ICMHR) |
| TELEPHONE/MHCMI |
| TELEPHONE SPINAL CORD INJURY |
| TELEPHONE/PRRC |
| TELEPHONE/PTSD |
| BLIND REHAB TELEPHONE |
| VIST TELEPHONE |
| TELEPHONE/DENTAL |
| TELEPHONE/DIALYSIS |
| TELEPHONE/ANESTHESIA |
| TELEPHONE/MH VOC ASSISTANCE |
| TELEPHONE RRTP |
| TELEPHONE/PROSTHETICS/ORTHOTICS |
| TELEPHONE/GEROPSYCHIATRICS |
| TELEPHONE POLYTRAUMA/Traumatic Brain Injury (TBI) |

**4: Cardiology and Primary Care Specific Outpatient Visit Days (either in-person or video-only) per 100 Veterans with Heart Failure Broken Down by Month from 2018-2020.**

**5: Cardiology and Primary Care Specific Video-Only Outpatient Visit Days per 100 Veterans with Heart Failure Broken Down by Month from 2018-2020.**

**6: Number and characteristics of Veterans for Analysis of Patterns of Outpatient Visits and Deaths for Veterans with Heart Failure between 2018 and 2020**

| **Year** | **2/1/2018 – 1/31/2019** | **2/1/2019 – 1/31/2020** | **2/1/2020 – 1/31/2021** |
| --- | --- | --- | --- |
| Number of Unique Veterans | 323,617 | 369,384 | 354,092 |
| Age (mean, SD) | 74.9 (10.78) | 74.05 (10.71) | 73.17 (10.50) |
| Male (n, %) | 313,617 (96.86) | 357,102 (96.68) | 341,594 (96.47) |
| Race (n, %) |  |  |  |
| White | 241,514 (74.63) | 274,686 (74.36) | 262,108 (74.02) |
| Black | 58,012 (17.93) | 66,913 (18.11) | 65,512 (18.50) |
| Other | 7,208 (2.23) | 8,443 (2.29) | 8,197 (2.31) |
| Missing | 16,883 (5.22) | 19,342 (5.24) | 18,275 (5.16) |
| Total deaths (n, %) | 55,075 (17.0) | 47,507 (12.9) | 58,284 (16.5) |
| Monthly mortality rates per **1000** Veterans (12 month mean, SD) | 12.86 (0.79) | 12.81 (0.83) | 13.08 (1.55) |
| Monthly all outpatient visit-days per **100** Veterans  (12-month mean, SD) | 81.36 (6.11) | 81.00 (5.63) | 57.80 (11.23) |
| Monthly in-person visit-days per **100** Veterans  (12-month mean, SD) | 78.27 (5.91) | 77.29 (5.43) | 47.16 (11.58) |
| Monthly video-only visit-days per **100** Veterans  (12-month mean, SD) | 3.09 (0.22) | 3.71 (0.40) | 10.64 (3.62) |
| Monthly telephone-only visit-days per **100** Veterans  (12-month mean, SD) | 128.43 (7.86) | 121.23 (6.44) | 167.27 (22.28) |
| Percent telephone visits lasting >20 minutes (% of total telephone visits) | 12.6% | 12.9% | 34.8% |

**7: Complete List of Baseline Characteristics (with list of comorbidities by Elixhauser comorbidity definitions) for the Association of Type of Outpatient Visits with Subsequent Mortality in Patients with Heart Failure**

| **Characteristics,**  **Mean (SD) or n (%)** | **No Visit in 2020** | **At least 1 in-person visit from February-September 2020** | **At least 1 video-only visit from February-September 2020** | **Telephone- only visit from February-September 2020** | **Total** |
| --- | --- | --- | --- | --- | --- |
|  | n=6,729 | n=224,046 | n=79,346 | n=11,318 | n=321,439 |
| Age, years | 77.7 (12.2) | 73.3 (10.4) | 74.8 (10.9) | 77.1 (11.5) | 73.5(10.5) |
| Gender |  |  |  |  |  |
| Male | 6,512  (96.8%) | 216,229 (96.5%) | 77,042 (97.1%) | 11,055 (97.7%) | 310,838 (96.7%) |
| Race |  |  |  |  |  |
| White | 4,966 (73.8%) | 165,654 (73.9%) | 60,139 (75.8%) | 8,659 (76.5%) | 239,418 (74.5%) |
| Black | 985  (14.6%) | 43,296  (19.3%) | 12,879 (16.2%) | 1,668 (14.7%) | 58,828 (18.3%) |
| Other | 167  (2.5%) | 4,886  (2.2%) | 1,835  (2.3%) | 223  (2.0%) | 7,111  (2.2%) |
| Missing | 611  (9.1%) | 10,210  (4.6%) | 4,493  (5.7%) | 768  (6.8%) | 16,082  (5.0%) |
| Married | 3,604  (53.6%) | 118,424 (52.9%) | 44,187 (55.7%) | 6,555 (57.9%) | 172,770 (53.7%) |
| Urban^ | 6,281 (93.3%) | 210,740 (94.1%) | 73,560 (92.7%) | 10,617 (93.8%) | 301,198 (93.7%) |
| VAMC Complexity ^^ |  |  |  |  |  |
| 1 | 5,460 (81.1%) | 188,850 (84.3%) | 63,798 (80.4%) | 9,385 (82.9%) | 267,493 (83.2%) |
| 2 | 591  (8.8%) | 16,199  (7.2%) | 6,499  (8.2%) | 913  (8.1%) | 24,202  (7.5%) |
| 3 | 678  (10.1%) | 18,997  (8.5%) | 9,049  (11.4%) | 1,020  (9.0%) | 29,744  (9.3%) |
| COVID-19 Positive Test within VA^#^ | 60  (0.9%) | 11,498  (5.1%) | 2,535  (3.2%) | 173  (1.5%) | 14,266  (4.4%) |
| Left ventricular Ejection Fraction |  |  |  |  |  |
| <40% | 1,759 (26.1%) | 66,207  (29.6%) | 23,415 (29.5%) | 3,292 (29.1%) | 94,673 (29.5%) |
| >=40% | 2,412 (35.8%) | 133,172 (59.4%) | 40,189 (50.7%) | 4,587 (40.5%) | 180,360 (56.1%) |
| Missing | 2,558 (38.0%) | 24,667  (11.0%) | 15,742 (19.8%) | 3,439 (30.4%) | 46,406 (14.4%) |
| BNP (pg/ml) | 948.5 (1420.7) | 686  (2751) | 640.8 (2879.1) | 621.4 (1162.4) | 679.1  (2728.7) |
| NT-proBNP (pg/ml) | 4,941.80 (8765.4) | 3,787.10 (9394.6) | 3,308.41 (6704.8) | 3,622.91 (6320.5) | 3,713.68 (8941.7) |
| Missing BNP or NTproBNP | 252  (3.7%) | 21,539  (9.6%) | 4,682  (5.9%) | 444  (3.9%) | 26,917  (8.4%) |
| Comorbidities^^^ |  |  |  |  |  |
| Alcohol Abuse | 304  (4.5%) | 18,757  (8.4%) | 4,538  (5.7%) | 485  (4.3%) | 24,084  (7.5%) |
| Autoimmune Conditions | 843  (12.5%) | 49,172  (21.9%) | 11,945 (15.1%) | 1,385 (12.2%) | 63,345 (19.7%) |
| Chronic Blood Loss Anemia | 48  (0.7%) | 3,996  (1.8%) | 777  (1.0%) | 78  (0.7%) | 4,899  (1.5%) |
| Leukemia | 30  (0.4%) | 1,995  (0.9%) | 487  (0.6%) | 53  (0.5%) | 2,565  (0.8%) |
| Lymphoma | 45  (0.7%) | 3,162  (1.4%) | 628  (0.8%) | 70  (0.6%) | 3,905  (1.2%) |
| Metastatic Cancer | 31  (0.5%) | 2,445  (1.1%) | 384  (0.5%) | 30  (0.3%) | 2,890  (0.9%) |
| Solid Tumor Without Metastasis, In Situ | 162  (2.4%) | 10,705  (4.8%) | 2,599  (3.3%) | 310  (2.7%) | 13,776  (4.3%) |
| Solid Tumor Without Metastasis, Malignant | 333  (4.9%) | 24,808  (11.1%) | 5,558  (7.0%) | 563  (5.0%) | 31,262  (9.7%) |
| Cerebrovascular Disease | 451  (6.7%) | 26,778  (12.0%) | 7,030  (8.9%) | 737  (6.5%) | 34,996 (10.9%) |
| Coagulopathy | 141  (2.1%) | 11,124  (5.0%) | 2,546  (3.2%) | 273  (2.4%) | 14,084  (4.4%) |
| Dementia | 478  (7.1%) | 11,504  (5.1%) | 3,351  (4.2%) | 460  (4.1%) | 15,793  (4.9%) |
| Depression | 712  (10.6%) | 53,425  (23.8%) | 11,273 (14.2%) | 1,074  (9.5%) | 66,484 (20.7%) |
| Diabetes | 1,955 (29.1%) | 116,741 (52.1%) | 34,833 (43.9%) | 3,783 (33.4%) | 157,312 (48.9%) |
| Drug Abuse | 165  (2.5%) | 11,626  (5.2%) | 2,165  (2.7%) | 243  (2.1%) | 14,199  (4.4%) |
| Hypertension | 3,633 (54.0%) | 180,217 (80.4%) | 57,109 (72.0%) | 7,142 (63.1%) | 248,101 (77.2%) |
| Liver Disease, Mild | 219  (3.3%) | 16,140  (7.2%) | 3,589  (4.5%) | 411  (3.6%) | 20,359  (6.3%) |
| Liver Disease, Moderate to Severe | 29  (0.4%) | 2,525  (1.1%) | 495  (0.6%) | 36  (0.3%) | 3,085  (1.0%) |
| Chronic Pulmonary Disease | 1,194 (17.7%) | 74,985  (33.5%) | 20,939 (26.4%) | 2,233 (19.7%) | 99,351 (30.9%) |
| Neurological Disorders Affecting Movement | 149  (2.2%) | 8,823  (3.9%) | 2,043  (2.6%) | 208  (1.8%) | 11,223  (3.5%) |
| Other Neurologic Disorders | 129  (1.9%) | 7,296  (3.3%) | 1,680  (2.1%) | 150  (1.3%) | 9,255  (2.9%) |
| Seizures and Epilepsy | 85  (1.3%) | 5,716  (2.6%) | 1,366  (1.7%) | 161  (1.4%) | 7,328  (2.3%) |
| Obesity | 735  (10.9%) | 60,557  (27.0%) | 15,506 (19.5%) | 1,559 (13.8%) | 78,357 (24.4%) |
| Paralysis | 41  (0.6%) | 3,413  (1.5%) | 647  (0.8%) | 64  (0.6%) | 4,165  (1.3%) |
| Peripheral Vascular Disease | 652  (9.7%) | 43,256  (19.3%) | 10,907 (13.7%) | 1,097  (9.7%) | 55,912 (17.4%) |
| Psychoses | 237  (3.5%) | 20,735  (9.3%) | 3,758  (4.7%) | 367  (3.2%) | 25,097  (7.8%) |
| Pulmonary Circulation Disease* | 140  (2.1%) | 11,685  (5.2%) | 2,540  (3.2%) | 262  (2.3%) | 14,627  (4.6%) |
| Renal Failure, Moderate* | 742  (11.0%) | 37,230  (16.6%) | 10,719 (13.5%) | 1,306 (11.5%) | 49,997 (15.6%) |
| Renal Failure, Severe* | 193  (2.9%) | 14,911  (6.7%) | 3,241  (4.1%) | 275  (2.4%) | 18,620  (5.8%) |
| Hypothyroidism | 513  (7.6%) | 25,557  (11.4%) | 7,864  (9.9%) | 1,033  (9.1%) | 34,967 (10.9%) |
| Other Thyroid Disorders | 52  (0.8%) | 5,760  (2.6%) | 1,233  (1.6%) | 110  (1.0%) | 7,155  (2.2%) |
| Peptic Ulcer with Bleeding | 33  (0.5%) | 3,084  (1.4%) | 752  (0.9%) | 64  (0.6%) | 3,933  (1.2%) |
| Valvular Heart Disease | 423  (6.3%) | 27,114  (12.1%) | 7,316  (9.2%) | 787  (7.0%) | 35,640 (11.1%) |
| Weight Loss | 190  (2.8%) | 10,033  (4.5%) | 2,434  (3.1%) | 256  (2.3%) | 12,913  (4.0%) |
| P-values < 0.001 for between group comparison of all covariates  ^ Urban setting determined using Rural-Urban Commuting Area (RUCA) codes from the Veterans’ census tract, where codes 1-6 (Metropolitan area) represent urban setting.  ^^The complexity VAMC model presented divides VHA facilities into 3 levels: level 1 represents high complexity, level 2 represents medium complexity, and level 3 represents low complexity, based on patient risk, levels of teaching/research activity, levels of ICU units, and number of Veterans Equitable Resource Allocation (VERA) pro-rated persons.  # COVID-19 positivity determined by either antigen or polymerase chain reaction (PCR) testing obtained within the VA system  ^^ All comorbidities defined by Elixhauser comorbidity definitions, determined using ICD10 codes from VA claims  * Determined by Elixhauser comorbidity definitions as:  1) Pulmonary Circulation Disease: primary pulmonary hypertension, kyphoscoliotic heart disease, other secondary pulmonary hypertension, unspecified pulmonary hypertension, secondary pulmonary arterial hypertension, pulmonary hypertension due to left heart disease, pulmonary hypertension due to lung diseases and hypoxia, chronic thromboembolic pulmonary hypertension, other secondary pulmonary hypertension, cor pulmonale (chronic), chronic pulmonary embolism, Eisenmenger’s syndrome, other specified pulmonary heart diseases, unspecified pulmonary heart disease, arteriovenous fistula of pulmonary vessels, aneurysm of pulmonary artery, other diseases of pulmonary vessels, unspecified disease of pulmonary vessels  2) Renal Failure, Moderate: chronic kidney disease stage 3 (moderate), chronic kidney disease stage 3 unspecified, chronic kidney disease stage 3a, chronic kidney disease stage 3b, unspecified chronic kidney disease, unspecified kidney failure  3) Renal Failure, Severe: Hypertensive chronic kidney disease with stage 5 chronic kidney disease or end stage renal disease, Hypertensive heart and chronic kidney disease without heart failure (with stage 5 chronic kidney disease or end stage renal disease), Hypertensive heart and chronic kidney disease with heart failure (with stage 5 chronic kidney disease or end stage renal disease), chronic kidney disease stage 4 (severe), chronic kidney disease stage 5, end stage renal disease, encounter for fitting and adjustment of extracorporeal dialysis catheter, encounter for fitting and adjustment of peritoneal dialysis catheter, encounter for adequacy testing for hemodialysis, encounter for adequacy testing for peritoneal dialysis, patient’s noncompliance with renal dialysis, kidney transplant status, dependence on renal dialysis | | | | | |

**8: Association of Type of Outpatient Visits with Subsequent Mortality in Patients with Heart Failure Using Historical Cohorts (2018 and 2019)**

**2019 Analysis**

| **TYPE OF VISIT (IN-PERSON, VIDEO-ONLY, OR TELEPHONE-ONLY) COMPARED WITH NO VISIT (n=196,910)** | |
| --- | --- |
| **Exposure in Model** | **Hazard Ratio (95% CI)** |
| At least one in-person visit day (n=173,899) vs. no visit (n=7,315) | 0.63 (0.58, 0.68) |
| At least one video-only visit day (n=1,617) vs. no visit (n=7,315) | 0.76 (0.64, 0.90) |

**2018 Analysis**

| **TYPE OF VISIT (IN-PERSON, VIDEO-ONLY, OR TELEPHONE-ONLY) COMPARED WITH NO VISIT (n=161,118)** | |
| --- | --- |
| **Exposure in Model** | **Hazard Ratio (95% CI)** |
| At least one in-person visit day (n=142,834) vs. no visit (n=4,935) | 0.39 (0.25, 0.63) |
| At least one video-only visit day (n=1,169) vs. no visit (n=4,935) | 0.48 (0.14, 1.63) |

**9: Sensitivity Analyses for the Association of Type of Outpatient Visits with Subsequent Mortality in Patients with Heart Failure Using a Blanking Period**

| **DAYS WITH ANY OUTPATIENT VISIT COMPARED WITH NO VISIT (n=290,789)** | |
| --- | --- |
| **Exposure in Model** | **Hazard Ratio (97.5% CI)** |
| Any outpatient visit day vs. no visit | 0.68 (0.62, 0.75) |
|  |  |
| **TYPE OF VISIT (IN-PERSON, VIDEO-ONLY, OR TELEPHONE-ONLY) COMPARED WITH NO VISIT (n=290,789)** | |
| **Exposure in Model** | **Hazard Ratio (97.5% CI)** |
| At least one in-person visit day vs. no visit | 0.65 (0.59, 0.72) |
| At least one video-only visit day vs. no visit | 0.71 (0.64, 0.78) |
| Telephone-only visit day vs. no visit | 0.76 (0.69, 0.87) |
| **AT LEAST ONE IN-PERSON VISIT DAY COMPARED WITH VIDEO-ONLY VISIT DAYS (N=276,161)** | |
| **Exposure in Model** | **Hazard Ratio (99% CI)** |
| At least one in-person visit day vs. video-only visit day | 0.92 (0.89, 0.96) |
|  |  |
| **IN-PERSON AND VIDEO-ONLY VISIT DAYS COMPARED WITH TELEPHONE-ONLY VISIT DAYS (n=285,926)** | |
| **Exposure in Model** | **Hazard Ratio (99% CI)** |
| At least one in-person visit day vs. telephone-only visit day | 0.84 (0.78, 0.92) |
| At least one video-only visit day vs. telephone-only visit day | 0.92 (0.84, 1.00) |

**10: Sensitivity Analyses for the Association of Type of Outpatient Visits with Subsequent Mortality in Patients with Heart Failure Including Days with Only Primary Care or Cardiology Outpatient Clinical Encounters**

| **DAYS WITH ANY CARDIOLOGY/PRIMARY CARE OUTPATIENT VISIT COMPARED WITH NO VISIT (n=321,439)** | |
| --- | --- |
| **Exposure in Model** | **Hazard Ratio (97.5% CI)** |
| Any cardiology or primary care visit day vs. no visit | 0.68 (0.66, 0.70) |
| **TYPE OF CARDIOLOGY/PRIMARY CARE VISIT (IN-PERSON OR VIDEO-ONLY) COMPARED WITH NO VISIT (n=321,439)** | |
| **Exposure in Model** | **Hazard Ratio (97.5% CI)** |
| At least one in-person visit day vs. no visit | 0.63 (0.61, 0.65) |
| At least one video-only visit day vs. no visit | 0.74 (0.72, 0.76) |
| **AT LEAST ONE IN-PERSON CARDIOLOGY/PRIMARY CARE VISIT DAY COMPARED WITH VIDEO-ONLY VISIT DAYS (N=276,529)** | |
| **Exposure in Model** | **Hazard Ratio (99% CI)** |
| At least one in-person outpatient visit day vs. video-only visit day | 0.85 (0.83, 0.87) |
|  |  |

Cox regression adjusted for age, sex, race, marital status, ejection fraction, COVID positivity, number of visits in prior temporal time (February 2019-September 2019), hospitalization, ED visits, Elixhauser comorbidities and hospital as fixed effects.
