# Supplementary material for: Outpatient care changes and associated mortality among Veterans with heart failure during the COVID-19 pandemic
Source: PLoS One. 2025 May 16;20(5):e0323308. doi: 10.1371/journal.pone.0323308 (PMC12083836; doi:10.1371/journal.pone.0323308)
Supplement: Attachment — Submitted filename: plos_one_6.3.24.docx [file pone.0323308.s002.docx]

Thank you for the opportunity to review this paper. Understanding how the shift to telehealth during COVID in the VA affected mortality is very important, particularly in the context of potential policy changes being debated at CMS. I have some comments for the authors below.

**Major comments:**

1. Please modify odds ratios to be marginal effects. See a recent publication Norton et al. on the challenges with interpreting odds ratios outside of case-control studies: <https://onlinelibrary.wiley.com/doi/10.1111/1475-6773.14337>
2. The authors write that “the main exposure was outpatient visit day…” I’m not sure what this means. Could the authors elaborate on how the exposure was calculated? Is it a binary variable indicator whether the patient had an outpatient visit at any time in the outcome period? Is it the number of visits? What level of observation is used here?
3. I have some concerns about the statistical analysis that the authors conducted
   a. It would be helpful to see an estimating equation. What is the outcome? What is on the right-hand side? It looks like the authors used both a Cox regression and a GEE? What is the reason for estimating both? Why would time to event be a useful measure here?
   b. It looks like the authors included VA health care use for one year from the baseline. Can the authors explain a little more what this looked like? Are they summing all visits *before* the pandemic period? A little clarification would be helpful. I’m hoping to verify that the authors did not include utilization *during* the pandemic period.
   c. How was the variable for positive COVID-19 test coded? Was that a binary indicator? Additionally, where was this information obtained? From VHA CDW?
   d. The authors write that they tested for effect modification by COVID-19 status and several other variables by fitting interaction terms. Because there are issues with interaction terms in non-linear models, these are difficult to interpret. I suggest the authors estimate these as stratified models instead.
4. I’m glad to see that the authors ran their models in 2018 and 2019 as well. Those results, in the appendix now, should be discussed somewhat in the context of their findings. The fact that there is a mortality difference in 2020 between in-person and other visit types seems consistent with the pattern in 2018 and 2019. Are there possibilities for patient selection? For instance, are high-need patients in rural areas with less appointment availability more likely to use telehealth or not receive a visit to begin with?
