# Supplementary material for: Outpatient care changes and associated mortality among Veterans with heart failure during the COVID-19 pandemic
Source: PLoS One. 2025 May 16;20(5):e0323308. doi: 10.1371/journal.pone.0323308 (PMC12083836; doi:10.1371/journal.pone.0323308)
Supplement: Attachment — Submitted filename: rebuttal letter.docx [file pone.0323308.s004.docx]

August 26^th^, 2024

*Rebuttal letter*

Dear Members of the Editorial Staff & Reviewers, PLoS One,

We are pleased to re-submit our manuscript entitled, “**Outpatient care changes and associated mortality among Veterans with heart failure during the COVID-19 pandemic**” for consideration for publication in *PLoS One*. We have carefully reviewed all comments made by the Editorial Board and Reviewers and revised our manuscript accordingly. Please see below for responses to the comments and suggestions made by the Editorial Board and Reviewers. All suggested changes have been made in the re-submitted manuscript.

**Reviewer #1: 
1) Table 1. In the legend, provide more detailed info re stat applied to determine, for example, stat differences across groups in pulmonary Vascular Disease. Legend only states P <....**

We apologize for the oversight. We have added the stats used for the comparison of baseline characteristics under the Statistical Analyses section of the Methods and in the footnote for Table 1: “Chi-square analyses were used for categorical and ANOVA for continuous variables.”

**2) Table 2. Excellent summary of the COX regression modeling.**

Thank you!!

**3) Figure 1. Difficult to delineate which line represents Total Outpatient Visits Febr/2020-ff.**

We have now added subheading labels for total outpatient visits and video only visits for the graph and included the figure on a separate file in a high quality figure.

**4) Figure 2. Discuss possible reasons that the apparent increase in mortality during Covid did not occur until November and forward of 2020.**

We thank the reviewer for noting this point. We have added in the second paragraph of the Discussion the potential reasons: “Although overall death rate had increased in the US during COVID-19 in 2020 [14], study of excess mortality in the VA population shows that the most significant increase happened in the 4^th^ quarter of 2020 [15], a pattern also shown in our study. Like other respiratory illnesses, COVID-19 has shown a tendency to decrease during summertime and increase again in fall and winter times. Further, we speculate that higher risk groups such as those with HF, may have been strictly isolated in the initial phase of the pandemic and may not have had significant exposure till later in the year. Despite the rise in mortality observed in the general US population related to the COVID-19 pandemic, the low (<5%) COVID-19 positivity rates in our study population of Veterans with HF suggest that COVID-19 infection may not have played a significant impact in the mortality risk of our population during the study period. Indeed, despite a significant uptrend in the slope of monthly mortality rates in the 2/2020-1/2021 period for our population compared to previous years, the annual average in monthly-mortality rates compared to the previous 2 years remained similar.”

**5) Expand discussions re distribution of established risk factors, e g BNP, across visit groups and possible impact on survival, in addition to pharma adherence.**

We thank the reviewer for this comment. We have now added a new paragraph (Discussion, para 4) : “It is important to note that the distribution of baseline characteristics across visit groups suggested that patients with in-person encounters were sicker as evidenced by a higher prevalence of comorbidities such as hypertension, diabetes, and chronic pulmonary disease, among other conditions compared to the rest, which would have put them at a higher risk of mortality. Conversely, we found the medication-possession-ratio for cardiovascular medications, to be highest for patients with in-person, followed by video-only, then telephone-only and lowest amongst patients with no-outpatient visits. Therefore, it is reasonable to hypothesize that the lower mortality risk associated with the in-person visit patient group may be related to the adherence to medications as a potential causal pathway to the association between type of encounter and mortality risk in the study population.”

**6) The Cox modeling adjusts for a large set of risk factors/co-variates, but there is no discussion as to their possible pathophysiological role of such risk factors/covariates in mediating/moderating the mortality outcome. It's especially interesting that there are no stat sign differences in biomarkers (although in dx) across visit groups in Table 1. Several of these biomarkers are closely related to HF outcomes, e.g., BNP.**

We included in the methods “These covariates were chosen since they are known or expected independent risk factors for mortality and potentially associated with presence and type of outpatient visit days and mortality in HF.” Given the large sample size in each of the groups stratified by type of visits, the comparisons of the different baseline characteristics, including BNP levels, were statistically significant between the groups. This was included as part of the legend in the footnote of Table 1. We have also incorporated the potential impact of the comorbidity distribution in mortality risk estimation in the discussion paragraph 4 (see response to comment #4), and the measures we have taken to account for those factors within the limitation paragraph of the discussion.

**7) Expand discussion re implication of decreased overall outpatient visits (although virtual (video and phone) visits have increase) during Covid. Is this a trend that holds true still? I don't request additional analysis. However, the authors mention the need for further studies of the optimal mix of in-person vs virtual visits. There is no data presented, as far as this reviewer can find, that outcomes are better than pre-Covid for any of the three visit types.**

The reviewer is correct that no published data is available (to our knowledge) of the comparison of volume of in-person and virtual visits during COVID vs. pre-COVID times. Indeed, available data in other type of cardiac visits such as the provision of cardiac rehabilitation for Medicare beneficiaries suggest that the encounter volume for cardiac rehabilitation continue to be lower than pre-pandemic times. To discuss these points, we have expanded on the additional implication of the decreased overall outpatient visits (although virtual [video and phone] visits have increased) during COVID times in the non-VA sector using cardiac rehabilitation as an example. We added these sentences to the discussion and have deleted the assertion that the mix of in-person and virtual visits were optimal since there is no data to support such statement. The revised paragraph reads (last para of discussion prior to limitations): “the decline in attendance to outpatient visits was not limited to the VA but extended throughout the US, including other lifesaving cardiac interventions, such as cardiac rehabilitation. This decline has led to closure of cardiac rehabilitation centers, a trend that persisted throughout the pandemic through September of 2022. Like our findings, the cardiac rehabilitation visit’s decline was insufficiently compensated by the utilization of virtual cardiac rehabilitation visits. As such, the availability of a virtual option in care interspersed to the in-person visits may be an option to maintain access to services during epidemic surges of respiratory illness or to overcome other barriers in care such as geographic distance or transportation difficulty. These results may provide the basis for the design of future trials to proactively re-design and optimize care modalities for the HF and other vulnerable patient populations, such as those with severe chronic obstructive pulmonary disease, cancer, diabetes mellitus on insulin-pump, among others.”

**8) When comparing mortality rates over time, how does rate look when only adjusting for pre vs peri Covid periods?**

We have now formally compared the average monthly mortality rates between the pre-COVID periods and the COVID period using T-tests and added a time series analysis to compare the change in monthly mortality rates over time between the pre-COVID and COVID periods (under statistical analyses in methods): “mean monthly visit and mortality rates between the pre-COVID periods were compared against the COVID period using T-tests. To determine whether a change over time occurred in monthly mortality rates, we used an interrupted time series analysis (ITSA) to compare the slope change, if any, in the monthly mortality rates between the pre-COVID periods (1/2018-1/2020) and the COVID period (2/2020-1/2021).”

In results, we added (second para): “the monthly unadjusted mortality rates were similar between study periods with an average monthly mortality of 12.9 (SD 0.8) in 2018-2019 and12.8 (SD 0.8) in 2019-2020, versus 13.1 (1.6) in 2020-2021 (P>0.60 for both comparisons of COVID vs. pre-COVID periods), per 1000 Veterans (Figure 2). However, time series analysis for temporal trends showed a significant uptrend in the slope for monthly mortality rates between the pre-COVID periods (2/2018-1/2020) versus the 2/2020-1/2021 (COVID period, P<0.01).

**9) Did VHA have sufficient virtual (video and phone) visits pre-Covid to allow for meaningful analysis.**

We were able to obtain sufficient pre-COVID data for meaningful analysis of the video visits but not telephone visits, since in pre-COVID times, telephone calls alone were not considered a valid clinical visit in the VA.

We stated: “Sensitivity analyses in the historical cohorts (2/2018-1/2019 and 2/2019-1/2020), excluding telephone-only visits in the analyses, showed similar estimates and results (supplement 8). Supplemental Table 8 contains the analyses for video visits:

**2/2019-1/2020 Analysis**

| **TYPE OF VISIT (IN-PERSON or VIDEO-ONLY) COMPARED WITH NO VISIT (n=196,910)** | |
| --- | --- |
| **Exposure in Model** | **Hazard Ratio (95% CI)** |
| At least one in-person visit day (n=173,899) vs. no visit (n=7,315) | 0.63 (0.58, 0.68) |
| At least one video-only visit day (n=1,617) vs. no visit (n=7,315) | 0.76 (0.64, 0.90) |

**2/2018-1/2019 Analysis**

| **TYPE OF VISIT (IN-PERSON or VIDEO-ONLY) COMPARED WITH NO VISIT (n=161,118)** | |
| --- | --- |
| **Exposure in Model** | **Hazard Ratio (95% CI)** |
| At least one in-person visit day (n=142,834) vs. no visit (n=4,935) | 0.39 (0.25, 0.63) |
| At least one video-only visit day (n=1,169) vs. no visit (n=4,935) | 0.48 (0.14, 1.63) |

We included under “Historical Cohorts” section, in Methods the following: “We excluded the telephone-only cohort in these historical cohort analyses, since telephone-only visits in pre-COVID times were not representative of medical visits”.

**10) Expand discussions as to the drivers of more virtual visits during Covid. Are there any indications that the most (multi) sick patients, and with the worst LVEF were more likely to avoid in-person healthcare visits? Table 1 does not show significant differences across several of the categories, e.g., urban vs rural that the authors discuss the VHA has addressed existing digital divide. Rather than merely listing the various programs the VHA has launched to address socioeconomic/racial and geographic digital divide, the authors should recognize the challenges with these programs to actually impact health-related divide.**

We thank the reviewer for this important point. Per the response to previous comments #5 and #6, we have added in the discussion section how there was a higher prevalence of sicker patients in the in-person visit group (all differences were statistically significant per table footnote) and how multi-variate regression analyses were able to account for the differential distribution of comorbidities and yet still found the in-person (sicker) visit group to have a reduced mortality risk compared to the virtual- or no-visit groups. We added the following to the limitation section of the discussion: “The differential distribution in age, race, and important comorbidities such as diabetes and COPD, and type of HF, that is observed between the different visit groups may contribute to the difference in mortality between the groups. We attempted to account for these confounders by performing extensive adjustment for demographic variables including marital status, comorbidity burden and health care utilization variables, characteristics of the HF such as BNP and LVEF, rurality, and COVID-19 test status. However, we acknowledge that residual confounding may persist and warrant consideration in interpreting the results. Factors such as perceived health status, ease of access to clinic or technology, and health literacy can potentially impact the type of outpatient visits and subsequent mortality but are not adequately captured..”

In terms of the digital divide we also added: “It is also important to note that that the VHA has taken systemic measures to minimize transportation and digital disparities, which may reduce some of the confounding related to ability to use technology….Despite the above, these programs often face numerous challenges such as patient acceptance or the availability of sufficient institutional resources, which may limit its impact on the health-related digital divide.”

**Reviewer #2: 
1. Please modify odds ratios to be marginal effects. See a recent publication Norton et al. on the challenges with interpreting odds ratios outside of case-control studies:** [**https://onlinelibrary.wiley.com/doi/10.1111/1475-6773.14337**](https://onlinelibrary.wiley.com/doi/10.1111/1475-6773.14337)

We agree with the reviewer of this important observation of showing marginal effects in lieu of odds ratios. We also agree with the concern raised in comment #3 below on whether a GEE modeling is needed to compare temporal trends in mortality rates, which produced odds ratios and have made the interpretation of the results complex and difficult. Reviewer 1 (comment #8) also suggested an unadjusted analysis of the change in mortality rates over time between pre-covid and covid periods. As such, we have decided to exclude the GEE analysis from the paper. Instead, we added simple T-tests for comparison of average monthly mortality rates between the pre-COVID and COVID periods and a time series analysis to compare the change over time (slope), if any, in monthly mortality rates between the pre-COVID and COVID periods for easy interpretation. To this effect, we have deleted the GEE analysis from the methods and results (including odds ratios) and added the following (under statistical analyses in methods): “mean monthly visit and mortality rates between the pre-COVID periods were compared against the COVID period using T-tests. To determine whether a change over time occurred in monthly mortality rates, we used an interrupted time series analysis (ITSA) to compare the change over time (slope change), if any, in the monthly mortality rates between the pre-COVID periods (1/2018-1/2020) and the COVID period (2/2020-1/2021).”

**2. The authors write that “the main exposure was** **outpatient visit day…” I’m not sure what this means. Could the authors elaborate on how the exposure was calculated? Is it a binary variable indicator whether the patient had an outpatient visit at any time in the outcome period? Is it the number of visits? What level of observation is used here?**

We apologize for the confusion; “outpatient visit day” refers to the number of days with outpatient encounters, a continuous variable. We chose “days” as opposed to “encounters” as the unit of exposure because patients may have multiple clinical encounters and of different types within the same day. In cases where multiple clinical encounters of different types (e.g. in-person, video and telephone) occurred within the same day, we used the encounter with the highest intensity of contact (in-person > video-only > telephone-only) as the visit type for the day. We have added the above text in the Exposure section of the Methods to further clarify the unit of exposure.

**3. I have some concerns about the statistical analysis that the authors conducted
a. It would be helpful to see an estimating equation. What is the outcome? What is on the right-hand side? It looks like the authors used both a Cox regression and a GEE? What is the reason for estimating both? Why would time to event be a useful measure here?**

Please see answer to comment #1 above regarding deleting the GEE model and instead using an interrupted time series analysis to compare the change in monthly mortality rates between the pre-COVID and COVID periods. We kept the Cox regression analysis to assess the relationship between Type-of-Outpatient Visit and Mortality and have added the following language in the statistical analysis section to clarify it: “We plotted Kaplan-Meier survival curves of HF patient groups stratified by presence of clinic days with: 1) in-person, 2) video-only, or 3) telephone-only encounters versus 4) the group with no-outpatient encounters. We related types of clinic encounters with mortality using Cox-regression models adjusting for potential demographic and clinical confounders.” We also added for clarity in the same section: “The index date is the first date of the exposure period (February 1, 2020) and patients were followed for all-cause mortality until May 31, 2021.”

**b. It looks like the authors included VA health care use for one year from the baseline. Can the authors explain a little more what this looked like? Are they summing all visits before the pandemic period? A little clarification would be helpful. I’m hoping to verify that the authors did not include utilization during the pandemic period.**

Correct, we have now clarified the **VA health care use for one year from the baseline** in the text as: “VHA healthcare use within one-year from the baseline (includes number of emergency-department visits, hospitalizations and hospice or palliative care visits) up to February 1, 2020.” It did not include utilization during the pandemic period which was operationally defined in our study as February 1, 2020.

**c. How was the variable for positive COVID-19 test coded? Was that a binary indicator? Additionally, where was this information obtained? From VHA CDW?**

The positive COVID-19 test was coded as a binary indicator. We modified the pre-existing text to: “positivity of COVID-19 test (by PCR or antigen test)” in the statistical analysis section of methods. The data was obtained from the VHA CDW. We added an additional phrase within the statistical analysis section methods to clarify this: “adjusting for potential demographic and clinical confounders obtained by cross-linkage of data between different databases within the VHA CDW (Corporate data warehouse)”.

**d. The authors write that they tested for effect modification by COVID-19 status and several other variables by fitting interaction terms. Because there are issues with interaction terms in non-linear models, these are difficult to interpret. I suggest the authors estimate these as stratified models instead.**

We thank the reviewer for this important comment. We agree that interactions terms may not adequately capture effect modification if the relationships are non-linear. Use of nonparametric approach such as stratified analysis is a good way to address this issue. In our study, the number of Veterans with COVID-19 in some of the comparator subgroups were very small (for non-visit group – only 60 people, and for telephone only group only 143 people). With our adjustment for nearly 40 variables to account for potential confounders, analysis in strata with these small sample sizes will likely not be informative. Since none of the tested interactions were significant, the amount of sensitivity analyses (historical cohorts in pre-COVID times and analysis with a blanking period excluding the exposure period for outcome accrual) and subgroup analyses that have already been conducted (by primary and cardiology encounters, excluding patients with no visits, excluding telephone visits, etc.), we will exclude the effect modification analyses from our methods and results in an effort to keep the paper focused on the main theme of comparison of type of outpatient visits and mortality,

**4. I’m glad to see that the authors ran their models in 2018 and 2019 as well. Those results, in the appendix now, should be discussed somewhat in the context of their findings. The fact that there is a mortality difference in 2020 between in-person and other visit types seems consistent with the pattern in 2018 and 2019. Are there possibilities for patient selection? For instance, are high-need patients in rural areas with less appointment availability more likely to use telehealth or not receive a visit to begin with?**

The reviewer raised an important point about the relationship between rurality and use of telehealth services. We have added the following sentence in the results section (third para): “Examination of the rurality of Veteran residence showed a relatively narrow range, where a high percentage of Veterans that utilized VA outpatient clinics resided in the urban settings, ranging from 92.7% for Veterans with video-only visits, 93.3% for Veterans with no visits, to 94.1% for Veterans with in-person visits.” We have also added the discussion of these results: “In our study, we found Veterans with no-visits and using video-only visits were more likely to have a rural residence, which supports the importance of virtual visits to maintain medical access rural Veterans. Nonetheless, the range of rurality across the study groups was relatively narrow within 1-2% point differences. This phenomenon is likely explained by the VHA policy that if the Veteran resides in an area too distant to a VHA clinic, they would likely be eligible for the VHA to pay for their medical care in the local community near their residence and would not have been included in the current study cohort.”

In terms of the comorbidity burden, the patients with higher burden of comorbidities were more likely to have in-person visits compared to the group of patients who only had video or telephone or no-visits. As such, we accounted for potential confounding by rurality or comorbidity burden by incorporating those variables in our Cox regression modeling.

**Reviewer #3:
Major:
1) My biggest concern is regarding the study design and the conclusions it allows one to draw; there are likely a number of unobservable confounders when comparing individuals who had no in-person or no video-based care over the exposure period to those who did, including frailty, beliefs about healthcare/the pandemic, and general engagement in care (particularly when comparing to those with no follow-up). This is noted in the limitations, but I worry that it significantly limits interpretation of these findings. In general, while the authors do this to some extent, it is important to avoid drawing causal conclusions from these results.**

We agree with the reviewer, hence have toned down our results and conclusions throughout the text and in the abstract to just report association. For example, the conclusion in the abstract and in the discussion now reads: “The presence and type of outpatient encounter was associated with the adjusted-risk of mortality for patients with HF during and before COVID periods.” We also emphasized in the discussion: “Future randomized-controlled studies are needed to causally evaluate whether different encounter types to provide care in HF patients affect their mortality outcomes.” We also re-emphasized the limitations of residual confounding in the limitation section of the discussion.

**2) What do you make of the fact that the same pattern of lower mortality risk with in-person and video-only visits compared to those with no outpatient visits was also observed in the pre-pandemic period (2018 and 2019)? A bit more discussion/interpretation of these findings would be helpful.**

We expanded the discussion of these findings and added the following sentences (third para, discussion) to provide a potential link between type of visit and outcomes, through adherence to medications as a surrogate for overall engagement in healthcare: “The distribution of baseline characteristics across visit type subgroups suggested that patients with in-person encounters were sicker, as evidenced by a higher prevalence of comorbidities such as hypertension, diabetes, and chronic pulmonary disease, among other conditions compared to the rest, which would have put them at a higher risk of mortality. Conversely, we found the medication-possession-ratio for cardiovascular medications, to be highest for patients with in-person, followed by video-only, then telephone-only and lowest amongst patients with no-outpatient visits. Therefore, we hypothesize that the lower mortality risks associated with patients with in-person visits may be related to the adherence to medications as a potential causal pathway… Additionally, medication-possession-ratio may also be a marker for the patient’s overall adherence to healthcare interventions and the results would suggest that those HF patients who were more adherent to health interventions were associated with better mortality outcomes.”

**Minor:
3) It would be helpful to the reader in the abstract and conclusion to note that while there was an increase in virtual visits as stated, this was only partially compensatory (i.e., overall follow-up was decreased during the pandemic compared to pre-pandemic period).**

We thank the reviewer and have added in the abstract and in the conclusion of the paper: “Despite an increase in video and use of telephone visits during the COVID-19 pandemic, there was still a decrease in total outpatient visits for patients with HF.”

**4) I may be misinterpreting, but for all the figures with visits per 100 Veterans, if the Y-axis is showing annual visit rates per 100 Veterans--wouldn't that mean that e.g., an annual rate of 80 visits per 100 Veterans = 0.8 annual visits, not 8?**

We apologize for this oversight – the figure legends have been corrected to reflect the average monthly visit is 81 per 100 and not 8.

**5) With regard to your statement that "the low (<5%) COVID-19 positivity rates in our study population suggest that COVID-19 infection may not have played an important role in the higher annual mortality rate seen in 2020"--given you are only capturing COVID-19 tests done in the VA system and the general difficulty of obtaining a test at that point in the pandemic, it seems likely that the actual rate of COVID-19 positivity was much higher than this (as you note in the limitations), which undermines your conclusion that disruption in outpatient care is what caused the increased mortality.**

We agree with the reviewer and have removed all language regarding increased mortality. We also added in results per request of reviewer 1 (comment #8): “the monthly unadjusted mortality rates were similar between study periods with an average monthly mortality of 12.9 (SD 0.8) in 2018-2019 and 12.8 (SD 0.8) in 2019-2020, versus 13.1 (1.6) in 2020-2021 (P>0.60 for both comparisons of COVID vs. pre-COVID periods), per 1000 Veterans (Figure 2). However, time series analysis for temporal trends showed a significant uptrend in the slope for monthly mortality rates between the pre-COVID periods (2/2018-1/2020) versus the 2/2020-1/2021 (COVID period, P<0.01).”

We also changed the phrasing in the discussion to state that our findings offered an insight into the impact of disruption in care for HF patients in the VHA system and potential opportunities for telehealth care models to bridge the gap in care. We also added in the discussion: “the low (<5%) COVID-19 positivity rates in our study population of Veterans with HF suggest that COVID-19 infection may not have played a significant impact in the mortality risk of our population during the study period.”

**6) Very minor, but in the following sentence, "Our results suggest that the disruption and change in outpatient care maybe related to the increase mortality in this vulnerable population", there should be a space between "may" and "be" and I believe it should say "increased" mortality with a "d".**

Based on the above comment #5, this sentence was deleted.

**7) On p. 15, "sever" should be "severe".**

The error has been corrected in the text. **8) It would be helpful to understand why you defined 1/31/2020 as the end of the pre-pandemic period, as most lock-downs and disruptions in care began a bit later.**

We have added a sentence in methods (under study sample and cohort assembly) that clarifies this point: “February 1, 2020 was chosen as the start of the COVID-19 pandemic period in our study based on the first declaration of COVID-19 as a public health emergency in the U.S. issued on January 31, 2020 under Section 319 of the Public Health Service Act.”

**9) The supplement states that "Telephone visits are not specified as cardiology/primary care"--in fact they are, but require accounting for both primary and secondary stop codes.**

We thank the reviewer for this clarification and the sentence has been deleted from the footnote of the table to avoid confusion.

On behalf of the co-authors, we sincerely thank the reviewers for their thoughtful insights which had made significant improvements to the paper.

Wen-Chih Wu, MD, MPH
