# Supplementary material for: Outpatient care changes and associated mortality among Veterans with heart failure during the COVID-19 pandemic
Source: PLoS One. 2025 May 16;20(5):e0323308. doi: 10.1371/journal.pone.0323308 (PMC12083836; doi:10.1371/journal.pone.0323308)
Supplement: Attachment — Submitted filename: rebuttal letter.2.docx [file pone.0323308.s005.docx]

October 10^th^, 2024

*Rebuttal letter*

Dear Members of the Editorial Staff & Reviewers, PLoS One,

We are pleased to re-submit our manuscript entitled, “**Outpatient care changes and associated mortality among Veterans with heart failure during the COVID-19 pandemic**” for consideration for publication in *PLoS One*. We have carefully reviewed all comments made by the Editorial Board and Reviewers and revised our manuscript accordingly. Please see below for responses to the comments and suggestions made by the Editorial Board and Reviewers. All suggested changes have been made in the re-submitted manuscript.

**Reviewer: 
1) I'm not sure why the authors decided to remove the GEE estimates. I certainly didn't intend for that, instead, I would have removed the time-to-event modeling. I asked the authors initially why time-to-event would be an interesting outcome here, but they didn't respond to that question. I think the GEE answers the more useful question of the overall relationship with mortality rather than how much time passed before death. My suggestion is to remove the Cox Regression and Kaplan Meier curves, and focus on the results from the GEE with marginal effects.**

R/ We apologize for mis-interpreting the comments. We have re-integrated the GEE model to the paper and reported the marginal effects as opposed to ORs to study the risk-adjusted relationship between the impact of the COVID pandemic and mortality in patients with HF overall.

We would also like to keep the Cox Regression and Kaplan Meier curves as it is studying a different question, which is the risk-adjusted relationship between type-of-outpatient visits (in-person, video, telephone, none) and mortality in patients with heart failure.

**2) The authors write that they performed interrupted time series analysis. It would be very helpful to see the interrupted time series figure (showing the slope change) to drive the point that they're making.**

R/ We have now added the interrupted time series figure that shows a slope change in monthly crude mortality rates, between the pre-COVID periods (2/2018-1/2020) and the COVID period (2/2020-1/2021) as a new Figure 3.

On behalf of the co-authors, we sincerely thank the reviewers for their thoughtful insights which had made significant improvements to the paper.

Wen-Chih Wu, MD, MPH
