# Supplementary material for: Outpatient care changes and associated mortality among Veterans with heart failure during the COVID-19 pandemic
Source: PLoS One. 2025 May 16;20(5):e0323308. doi: 10.1371/journal.pone.0323308 (PMC12083836; doi:10.1371/journal.pone.0323308)
Supplement: Attachment — Submitted filename: rebuttal letter.3.1..docx [file pone.0323308.s006.docx]

February 26, 2025

*Rebuttal letter*

Dear Members of the Editorial Staff & Reviewers, PLoS One,

We are pleased to re-submit our manuscript entitled, “**Outpatient care changes and associated mortality among Veterans with heart failure during the COVID-19 pandemic**” for consideration for publication in *PLoS One*. We have carefully reviewed all comments made by the Editorial Board and Reviewers and revised our manuscript accordingly. Please see below for responses to the comments and suggestions made by the Editorial Board and Reviewers. All suggested changes have been made in the re-submitted manuscript.

**Reviewer: 
1) Please provide info in Abstract and in the Result section re p value (trend) when presenting the decreased likelihood for death in each of the three categories (1. at least one in-person visit. 2. Video only. 3. Telephone only) with ref categories no visits. OR and Conf Intervals are provided for each of the three categories. However, no stat data is provided across the three categories.**

R/ we have now added the p value for linearity trend of the adjusted log hazards for mortality from in-person, video-only, to telephone-only visits in both the results section and the abstract.

On behalf of the co-authors, we sincerely thank the reviewer for the thoughtful insight.

Wen-Chih Wu, MD, MPH
